# Knowledge, awareness and perception about equine glanders among veterinarians and medical professionals in India

**DOI:** 10.3389/fvets.2024.1334485

**Published:** 2024-03-14

**Authors:** Ana Raj, Anubha Pathak, Shanmugasundaram Karuppusamy, Bhupendra Nath Tripathi, Hema Tripathi, Harisankar Singha

**Affiliations:** ^1^ICAR-National Research Centre on Equines, Hisar, India; ^2^Faculty of Animal Sciences, Sher-e Kashmir University of Agricultural Sciences and Technology, Jammu, India; ^3^Krishi Anushandhan Bhawan-II, New Delhi, India

**Keywords:** *Burkholderia mallei*, glanders, equines, awareness, control, Knowledge

## Abstract

Glanders is a highly infectious and notifiable disease of equines that occurs due to *Burkholderia mallei*. In India, glanders re-emerged in 2006 and thereafter regular outbreaks have been reported in various states (*n* = 14). Frequent and prolonged contact with equids with glanders may transmit *B. mallei* infection to humans. This study was designed to learn more about the Knowledge, Awareness and Perception (KAP) of veterinarians, para veterinarians, and physicians about equine glanders, which will help in enhancing the nation-wide glanders eradication programme. A total of 165 respondent’s from 11 Indian states and one union territory were surveyed. Most of the respondents (*n* = 160) were from equine glanders affected or endemic states. Knowledge gap analysis revealed that 40.3 and 22% of the participants were not aware of government regulations and the transmission of glanders, respectively. These are major concerns given the wide spread occurrence of disease in the country. Awareness test on glanders revealed that 65(39.4%) participants would collect biological samples for laboratory confirmation, 67(40.6%) would inform the concerned authorities and 106 (64.2%) replied that they would eliminate the glanders infected equines. Analysis of perception towards equine glanders showed that majority of the participants (*n* = 113, 68.4%) observed that equine keepers were reluctant to disclose the clinical symptoms of *B. mallei* infection. Furthermore, non-co-operation and unwillingness by superiors (33.9%), financial (31%), administrative (28.4%), and technical limitations (27.8%) were major constraints under the perception analysis. This study reveals that veterinarians need to be educated on governmental policies and guidelines on equine glanders with regular training and awareness programs. Intersectoral co-ordination to investigate human glanders is also needed.

## 1 Introduction

Glanders is one of the notifiable equine diseases under the Prevention and Control of Infectious and Contagious Diseases in Animals Act, 2009 of Government of India. It is caused by *Burkholderia mallei*, a Gram-negative, non-spore forming, non-motile and facultative intracellular bacterium (1). *B. mallei* primarily infects donkeys, mules and horses. Nonetheless, other species such as camels, felines, small ruminants and carnivores are also susceptible to *B. mallei* infection (2). Ingestion of feed and water contaminated with *B. mallei* organism or entry of the bacterium through abrasive skin from the glanderous equids is a major route of disease transmission (1). Infection can also be transmitted through contaminated fomites as well (3). Generally, three clinical forms of glanders, namely, nasal, pulmonary and cutaneous or farcy are observed in equines. These forms are characterized by ulcerating lesions of the skin and mucous membranes, enlargement and hardening of lymphatic vessels, respiratory distress, nasal discharges, emaciation, septicaemia and death (4). In horses, the disease is chronic, whereas in donkeys it is acute, and in mules, it may be acute or chronic in nature. Chronically infected a symptomatic horses act as a source of infection to susceptible animals (5).

Frequent and prolonged contact with glanderous equids may transmit *B. mallei* infection to humans, such as veterinarians and para-veterinarians, equine handlers, and slaughterhouse workers and laboratory personnel (6, 7). Specific treatments and vaccines for animals and humans are not available against the *B. mallei* infection (8). Developed nations implemented stringent control measures and eliminated glanders in the middle of the 1900s after realising the organism’s potential as a bio-weapon during World Wars I and II and its high disease prevalence (9). However, glanders is still prevalent in certain parts of the world like South America, parts of Africa, the Middle East, Eastern, Central and Southern Asia (10). Over the past 15 years, a greater number of equine glanders with increasing frequency were reported in several countries, including United Arab Emirates, Kuwait, Lebanon, Pakistan, Brazil, Bangladesh, Nepal, Vietnam, China, Korea, Russia, Mongolia, Iraq, Iran and Bahrain (8, 11–13).

In India, glanders re-emerged in the state of Maharashtra in 2006, with 26 equines tested positive for equine glanders. Thereafter, glanders have been reported from several states of the country, such as Andhra Pradesh, Himachal Pradesh, Haryana, Punjab, Uttar Pradesh, and Uttarakhand (14). Between April 2011 and December 2014, a total of 7,794 equid sera samples from 10 Indian states were tested for equine glanders, of which 36 equines were sero-positive for glanders in 3 states such as Uttar Pradesh, Himachal Pradesh, and Chhattisgarh (15). Another sero-surveillance study from January 2015 to December 2018 tested 102,071equid sera samples from 22 Indian states and one union territory and reported 932 glanders-positive cases from12 states with an overall seroprevalence range between 0.62 and 1.145% in the country (4). Altogether, glanders surveillance from 2006 to 2018 revealed that glanders is prevalent in 14 states in India (4).

The Department of Animal Husbandry and Dairying, Government of India, has made advances in glanders surveillance and launched the National Action Plan on glanders in 2019. However, regular outbreaks pose many challenges to veterinarians, equine owners, public health workers, and policy makers in determining appropriate actions to prevent disease spread, control, and eradication. The Indian Livestock Census 2019 reports that there are approximately 0.5 million equines in the country. Equines have significant role in construction works, tourism, safari, public transportation, commodities transportation, carting, equestrian events, police and military/paramilitary forces. Awareness about equine and human glanders among veterinarians, para-veterinarians and physicians is crucial for the better implementation of glanders eradication program in the country. Therefore, we have conducted a Knowledge, Awareness and Perception (KAP) survey among veterinarians and physicians about equine glanders in order to identify knowledge gaps in disease surveillance, diagnosis, reporting and control.

## 2 Materials and methods

### 2.1 Study area and participants

In order to sensitize and to create awareness among veterinarians and para-veterinarians, Maharashtra, Uttar Pradesh and Madhya Pradesh state animal husbandry departments conducted workshops on glanders diagnosis and control. The ICAR-National Research Centre on Equines (ICAR-NRCE), Hisar, Haryana, collaborated with the state animal husbandry departments to organize these training programmes for veterinarians, physicians and microbiologists on equine glanders. Participants of these workshops and training programmes were considered for this study. At the end of these workshops/ training programmes, the participants were encouraged to participate in the survey. After explaining the purpose of the study and the design of questionnaire, survey forms were distributed to the interested participants. A total of 165 respondent’s from11 Indian states (Bihar, Chhattisgarh, Haryana, Jammu and Kashmir, Kerala, Maharashtra, Madhya Pradesh, Punjab, Rajasthan, and Uttar Pradesh) and one union territory (Delhi) responded voluntarily and submitted the filled-in questionnaires. The study was conducted from December 2018 to November 2021and the respondents’ consent was obtained to publish their data.

### 2.2 Questionnaire design

A structured questionnaire was framed for data collection and the questionnaire consisted of profile characteristics of respondents, knowledge, awareness and perception on equine glanders. In the profile characteristics section (section – 1), socio-demographic characteristics of the respondent like name, age, educational qualification, occupational status and place of work were collected so that they could be correlated with their knowledge level on equine glanders. Nominal and ordinal scales were used for this measurement. The knowledge test on glanders section (section – 2) was framed to assess the knowledge level of respondents on equine glanders and its control measures. This section was designed with 8 binary questions (yes or no questions), 2 incomplete statements and 10 multiple choice questions. Knowledge of respondents on 5 dimensions were studied namely basic nature of glanders (4 questions), key attributes of glanders (5 questions), transmission of glanders (2 questions), government regulations on glanders (5 questions) and glanders control measures (4 questions). To develop suitable training programmes and regulations for glanders control, it is necessary to assess the respondents’ level of awareness towards glanders.

Therefore, in the awareness section (section – 3), awareness statements on line of action during glanders outbreak and type of biological samples to be collected for glanders diagnosis was incorporated. Perception is the process by which an individual interprets and organize sensation to produce a meaningful experience (16). Perception studies were conducted to understand whether the veterinarians were well informed about the present state of affairs in glanders disease control. Under the perception section (section – 4), perceived factors behind poor control of glanders were studied by requesting respondents to list important factors. Besides, questions were framed to understand the constraints faced by the respondents in glanders management and the suggestions to overcome them (section – 5). Thus, the questionnaire was standardized with 5 sections and reviewed by subject matter experts for refinement. With minor changes in choice of words, the finalized questionnaire (Supplementary Data Sheet S1) was distributed among the targeted respondents.

### 2.3 Data collection

Convenience sampling (non-probability sampling) was adopted for executing this study as it was assumed that individuals who are dealing with equines directly or indirectly will participate in the workshop/ training programmes organized by ICAR-NRCE, Hisar. The purpose of the study was explained to the participants and they were instructed to attend all items provided in the questionnaire and were given enough time for completion. The data was collected and tabulated to determine the knowledge, awareness and perception of the respondents along with their suggestions to improve awareness of glanders among relevant stakeholders.

### 2.4 Statistical analysis

Descriptive statistics was employed to map the profile characteristics of the respondents. To study the knowledge level, the expected knowledge score under each of the 5-knowledge dimension was computed followed by the observed knowledge score. These scores were used in the calculation of knowledge level with the given formula. From the knowledge level formula, knowledge gap was determined for each knowledge dimension and ranked in descending-order. Statistical Package for Social Sciences (SPSS) 16.0 was utilized to correlate the knowledge level of the respondents in comparison to their profile characteristics. Spearman’s rank correlation was run to determine the relationship between profile characteristics of the respondents and their knowledge level. If the *p*-value is less than or equal to 0.05 (single asterisk), the Spearman correlation coefficient (ρ) is considered as statistically significant at 5% level of significance. Awareness and perception of the respondents were analyzed by percentage analysis.


$$\text{Knowledge}\;\text{level}\;\left(\%\right)=\frac{\text{Total}\;\text{observed}\;\text{knowledge}\;\text{scores}}{\text{Total}\;\text{expected}\;\text{knowledge}\;\text{scores}}x\;100$$

## 3 Result

### 3.1 Profile characteristics of respondents

**Figure 1:**
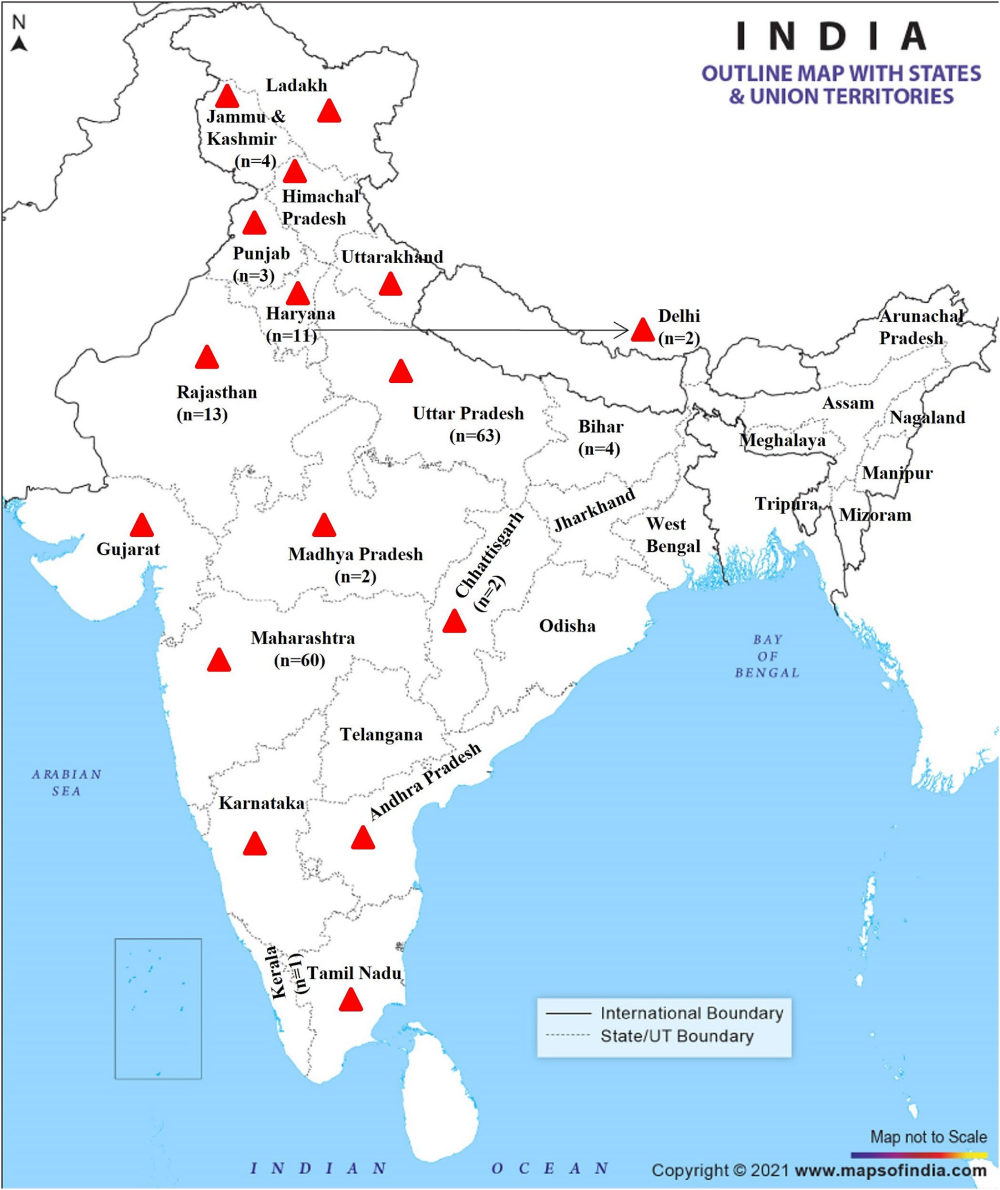
Map of India of showing equine glanders reported states (red triangle) and non-reported states; the number of respondents participated in survey from various states. Reproduced with permission.

In total, 165 respondents voluntarily participated in this survey, of whom141 (85.4%) were male and 24 (14.5%) were female (Table 1). Around 15 participants did not respond to the survey owing to training/ workshop-related obligations unrelated to this survey. As it was a cross-sectional study, data was collected from different individuals at a single point in time to describe the existing status of glanders disease awareness. Most of the respondents (*n* = 160) were from equine glanders affected or endemic states (Figure 1; Supplementary Table S1). Among these, 63 (38.2%) participants were from Uttar Pradesh, where equine glanders is highly endemic. Whereas, a total of 60 (36.4%) respondents were from Maharashtra; besides, significant numbers of participants were from Rajasthan (*n* = 13) and Haryana (*n* = 11). The number of participants surveyed in this study was less from Bihar (*n* = 4) and Kerala (*n* = 1) where glanders status is still unknown (Supplementary Table S1). Regarding the profession, 149 veterinarians, 12 physicians, and 4 para-veterinarians participated in the survey. The average experience of working in the field ranged from less than 5 years to more than 30 years, with median of 14 years (Table 1).

**Table 1:** Sociodemographic characteristics of respondents.

S. No.	Profile characteristics	Total number of participants (N = 165)	
		Frequency	Percentage (%)
**1.**	**Sex (N = 165)**		
	Male	141	85.45
	Female	24	14.54
**2.**	**Educational status (N = 165)**		
	Diploma	4	2.42
	Under graduation in veterinary sciences	62	37.57
	Under graduation in medicine and surgery	5	3.03
	Post-graduation in veterinary sciences	76	46.06
	Post-graduation in medicine and surgery	7	4.24
	Doctoral degree	11	6.66
	*Median –Post-graduate in veterinary sciences* *Minimum – Diploma holder* *Maximum – Doctoral degree in veterinary sciences* *Inter quartile Range – Under graduate in veterinary sciences*		
**3.**	**Occupational status (N = 165)**		
	Internship	10	6.06
	Livestock Development Officer (LDO)	47	28.48
	Assistant professors/Microbiologist/Pathologist/Epidemiologist/Veterinary Assistant Surgeon	25	15.15
	Veterinary Surgeon/Veterinary Medical Officer/ Veterinary Officer	9	5.45
	Assistant Director	44	26.66
	Deputy Director	26	15.75
	Joint Director	4	2.42
	*Median –Assistant Director* *Minimum –Internship* *Maximum – Joint Director* *Interquartile Range –Asst. Professor/ Microbiologist/ Pathologist/ Epidemiologist/ VAS*		
**4.**	**Length of service (N = 165)**		
	Less than 5 years	31	18.78
	6 to10 years	33	20
	11 to15 years	29	17.57
	16 to 20 years	19	11.51
	21to 25 years	28	16.96
	25 to30 years	14	8.48
	More than 35 years	11	6.66
	*Median – 14 years* *Minimum – 1 year* *Maximum – 34 years* *Interquartile Range − 15 years*		
	Morethan30 years	11	6.66

### 3.2 Knowledge level on glanders

**Figure 2:**
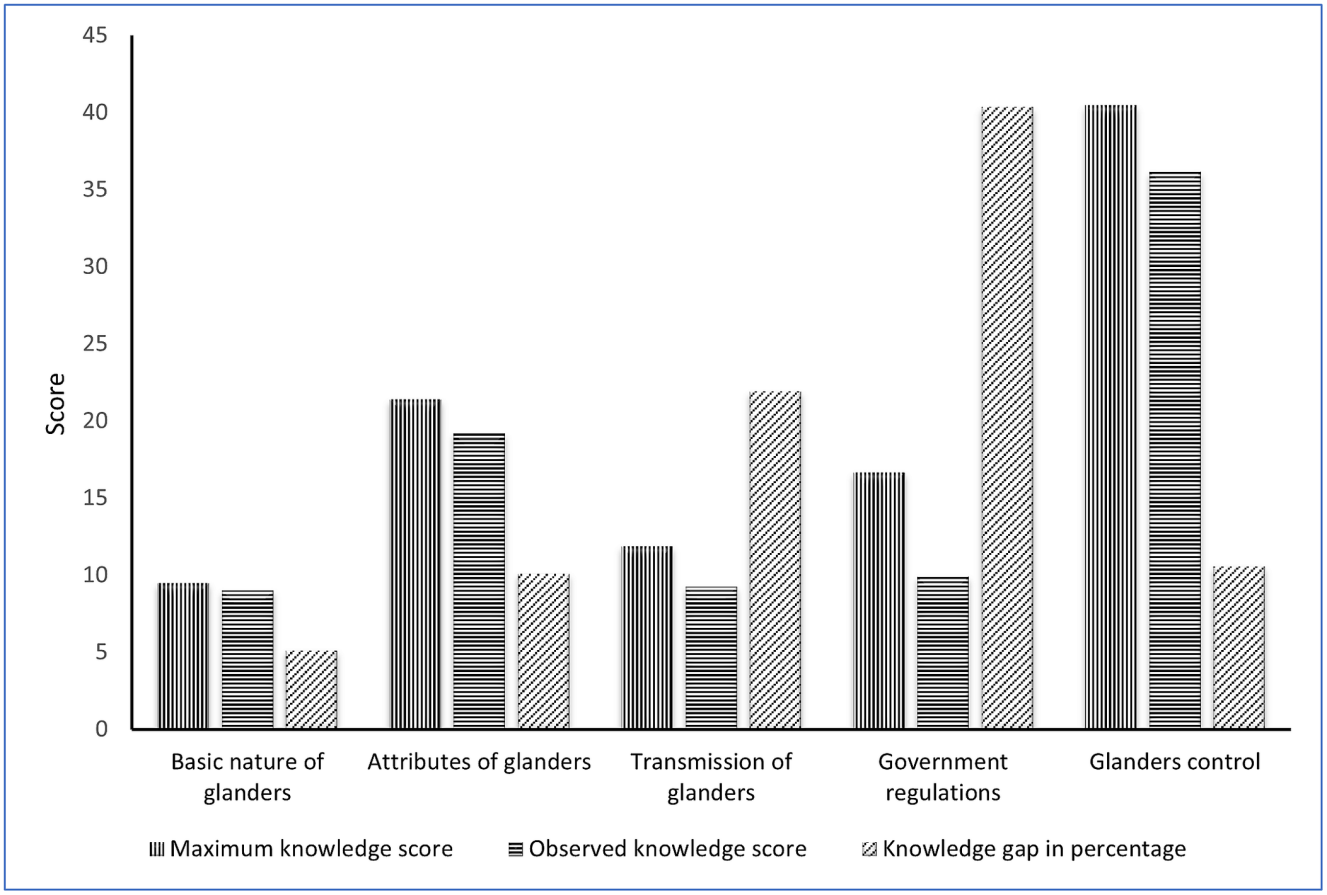
Knowledge gap of the respondents on equine glanders.

In knowledge gap analysis, participants were asked to provide their responses on various parameters on glanders, namely, the basic nature of glanders, attributes of glanders, transmission of glanders, government regulations, and glanders control activities, and their responses have been summarized in Table 2 and Figure 2. The respondent’s knowledge of equine glanders varied with an overall observed knowledge level of 83.7% and a knowledge gap of 16.3%. The observed knowledge level revealed that 36.2, 19.2 and 9% of the respondents were familiar with glanders control activities, attributes of glanders, and the basic nature of glanders, respectively (Figure 2). On the other hand, 40.3 and 22% of the participants were not aware of government regulations and the transmission of glanders, respectively, which have been found to be major concerns in knowledge gap analysis (Figure 2; Table 2). Analysis of knowledge of the respondents about the organization of equine fairs during disease outbreak showed that 99 (60%) participants were well-informed that the organizations of equine fairs during the glanders outbreak are not expected. However, 66 (40%) participants responded that the organization of equine fairs during glanders outbreaks is acceptable. A total of 75 (45.5%) participants have seen equine glanders cases in the field (Supplementary Table S2). The distribution of respondents based on their glanders knowledge level revealed that 21 (12.7%), 113 (68.5%) and 31 (18.8%) respondents have low, medium, and high levels of knowledge on glanders, respectively (Supplementary Table S2).

**Table 2:** Knowledge gap of respondents on glanders.

S. No.	Knowledge parameters	Expected knowledge scores	Observed knowledge scores	Knowledge level (%)	Knowledge gap (%)	Rank
1.	Basic nature of glanders	9.52	9.03	94.80	5.15	V
2.	Attributes of glanders	21.40	19.24	89.90	10.10	IV
3.	Transmission of glanders	11.90	9.29	78.06	21.94	II
4.	Government regulations	16.66	9.94	60	40.34	I
5.	Glanders control	40.47	36.19	89.42	10.58	III
	**Total**	**100**	**83.69**		**16.31**	

### 3.3 Awareness on equine glanders

Awareness test assessed the pro-activeness and preparedness of respondents while dealing with glanders outbreak. There were two opinion-based questions in this section for which the responses varied across the respondents. They were asked what they would do if they suspect a glanders outbreak; a total of 65 (39.4%) responded that they would collect biological samples for laboratory confirmation. After confirmation, 67 (40.6%) respondents said they would inform the concerned authorities for further necessary action, and 106 (64.2%) participants replied that they would eliminate the glanderous equids (Table 3). Interestingly, 13.3% (*n* = 22) of the participants replied that they Would quarantine the animal and initiate treatment (Table 3). The participants were asked to give their response on the type of biological samples that needed to be collected for glanders diagnosis, and multiple responses have been obtained (Supplementary Table S2). Blood, nasal swab and nodule swab samples were preferred by 150 (90.9%), 129 (78.2%) and123 (74.5%) respondents, respectively. However, 57.6% (*n* = 95) of the respondents answered that faecal and urine samples were suitable for glanders diagnosis.

**Table 3:** Line of action during glanders outbreak.

S. No.	Line of action	Frequency*	Percentage
1.	Inform the concerned authorities	67	40.60
2.	Collect samples and sent to lab for confirmation	65	39.39
3.	Quarantine the animal and initiate treatment	22	13.33
4.	Elimination of the animal if confirmed	106	64.24

*Multiple responses.

### 3.4 Perception towards equine glanders

Respondents were asked to state the perceived factors behind poor reporting and control of glanders in India. It was found that the majority of respondents (*n* = 113, 68.5%) replied that equine keepers were reluctant to disclose the clinical symptoms of *B. mallei* infection (Table 4). In addition, participants were asked to give their opinion about other constraints related to the reporting of glanders. A total of 56 (33.9%) respondents stated that superiors’ unwillingness to cooperate and report a glanders outbreak is a major constraint. In addition, 10.9% (*n* = 18) of the participants replied that glanders is not a priority disease in their area of posting. Financial (*n* = 51, 30.9%), administrative (*n* = 47, 28.5%), and technical (*n* = 46, 27.9%) constraints (Supplementary Table S2) also played a vital role in the underreporting of glanders outbreaks. Spearman’s correlation of independent variables with knowledge level revealed a strong positive relationship between occupational status and the knowledge level of an individual respondent, which was statistically significant (*p* = 0.016) (Table 5). This segment highlights the importance of sensitizing the freshly recruited animal health workers through workshops and or training would be helpful in glanders reporting, prevention and control.

**Table 4:** Factors behind poor control of glanders.

S. No.	Perceived factors	Frequency*	Percentage
1.	Glanders is not a priority disease in my locality	18	10.90
2.	Lack of focus or seriousness of field veterinarians towards glanders	20	12.12
3.	Owners hesitate to disclose the symptoms	113	68.48
4.	Lack of knowledge among veterinarians regarding glanders	30	18.18
5.	Non-cooperation and unwillingness by the superiors	56	33.93

*Multiple responses.

**Table 5:** Spearman’s correlation table of independent variables with knowledge level.

Variables	Spearman’s correlation coefficient (ρ)	*p* value
Place of posting	0.150	0.054
Educational level	0.101	0.195
**Occupational status**	0.187*	**0.016**
Length of service	0.022	0.778

*Significant at 5% level of significance.

## 4 Discussion

In our previous surveillance study, we have identified that limited knowledge and awareness of veterinarians and equine keepers are one of the major risk factors responsible for the prevalence of glanders in India (17). Therefore, this study was focused to assess veterinarians’ knowledge, awareness, and perception (KAP) about equine glanders. As per our knowledge, this is the first of its kind KAP study on glanders in India and elsewhere. The sampled participants (*n* = 165) were from 11 states, of which a larger number (*n* = 160) were from glanders endemic states. Inclusion of participants from the glanders endemic states would provide a better KAP survey because respondents who had previously encountered disease outbreaks would be more knowledgeable and in a better position to deal with the outbreak and post-outbreak management practices. For instance, a study of veterinarians’ awareness and perception towards leptospirosis revealed that veterinarians who practiced in the outbreak-reported areas were more knowledgeable on zoonoses, disease diagnosis, reporting, treatment and vaccination than those in leptospirosis non-reported counties (18). The average experience of participants ranged from less than 5 years to more than 30 years, with a median of 14 years. The participant’s length of service had a positive correlation with their knowledge of equine glanders and similar results have been found in other studies such as malaria and dengue (19).

One of the encouraging findings was that respondents were familiar with glanders control activities, attributes of glanders, and the basic nature of glanders. However, lack of awareness about government regulations was found to be a major concern in the knowledge gap analysis, given the widespread occurrence of disease in the country. This indicates that there is a need to educate the veterinary and medical professionals on various clauses described in the glanders control policy^1^ pertaining to mandatory reporting, elimination of the positive reactors, post-outbreak surveillance in the notified zone, biosafety, and bio-security measures. Furthermore, our study also found that 21.9% of respondents had a significant knowledge gap regarding disease transmission. Risk factors associated with the spread and maintenance of glanders are sharing of husbandry tools, drinking water, grazing sites and poor management practices. The secretions from the glanderous equids may contaminate the water and grazing areas and then infect other in-contact animals (2). Additionally, the spread of the infection is accelerated by the mobility of a symptomatic carriers without adequate screening (20). Thus, we emphasize that the veterinarians and equine keepers need to be educated on the factors involved in the spread of disease among the equids in order to prevent and control disease spread.

In this study, we have observed that 40% of participants responded that the organization of equine fairs during a glanders outbreak is acceptable. Though, the organization of equine fairs, congregations, and any equestrian events is not recommended within a 25-km radius of the notified area or focus of the infected zone as per the national action plan for control and eradication of glanders in India (2019). This revealed an immense lack of understanding and awareness among the respondents about the contagious nature and transmission of glanders. To address this issue, meetings with different stakeholders prior to any equestrian event may be helpful for the concerned authorities to take preventive measures to control disease outbreaks and spread. The distribution of respondents based on their glanders knowledge level revealed that 21 (12.7%) and 113 (68.5%) respondents have low and medium levels of glanders knowledge, respectively. This suggests that there is a need for regular training and capacity building on equine and human glanders for field veterinarians, para-veterinarians, and health officials involved in disease investigation and surveillance activities.

In this study, the majority of the respondents (68.5%) answered that the owners were reluctant to disclose the clinical symptoms of *B. mallei*, and this may be due to a lack of knowledge about the clinical manifestation of the disease among equine keepers. In addition, veterinarians who are not aware of the government policies will not be able to educate the equine keepers about the compensation amount, quarantine policies, zoonotic nature and importance of regular surveillance of glanders. An administrative delay in dispersion of compensation and inadequate compensation could be other factors for the non-disclosure of disease by equine keepers.

In India, as per the Glanders and Farcy Act, 1899, glanders-test positive equids to be euthanized and their owners need to be compensated with only 50 rupees (~1USD), which was continued till 2015. This compensation has been increased to Rs 25,000 (~300USD) per horse and Rs 16,000 (~250USD) per mule or donkey in 2015. An important step toward encouraging equine keepers to alter their behavior from “unwilling “to” proactive” participants would be the regular revision of compensation to a suitable amount and simplification of the administrative processes for speedy disbursement of the compensation (4). Though there is a clause in the national action plan on glanders to revise the compensation in every three years, it has not been followed so far, and it needs immediate attention from the policy makers. In Canada, equine glanders were eradicated by providing effective compensation to equine keepers. This was a tiny price paid to stop losses to the horse industry and to end the significant zoonotic threat posed to human health (21). Furthermore, targeted extension activities for health workers who regularly interact with equine keepers, their up-gradation of knowledge and equine keepers’ interaction training may also help in controlling then on-disclosure of glanders cases by equine keepers.

Perception of respondents towards equine glanders revealed major constraints such as non-co-operation and unwillingness by the superiors (33.9%), followed by financial (30.9%), administrative (28.58%), and technical glitches (28.97%). Non-cooperation and unwillingness by the superiors may be due to the subsequent additional activities associated with outbreak declaration, like disease notification, quarantine of the infected and in-contact animals, euthanasia of positive cases, processing of files for release of compensation, post-outbreak surveillance, carcass disposal, and enforcing zoo-sanitary measures. At the field level, the state animal husbandry department mainly pays more attention to infectious diseases of other livestock species, such as Foot and Mouth Disease, Brucella, Lumpy Skin Disease, Classical Swine Fever, African Swine Fever, Avian Influenza, etc., and neglects equine diseases (4). These could be the major reasons behind the unwillingness of the superiors and the major constraints in glanders surveillance, control, and prevention.

Financial constraints result from a lack of sufficient funds from state animal husbandry departments for the glanders control programme and less compensation money in comparison to the value of the equines. Administrative constraints may be due to geographical demarcation of the infected area, supervision of the local veterinary authority, difficulty in tracking animal movement, prohibition of equine mobility, complete screening of remaining equids within 3 weeks of the incidence of the first case, and repeated twice within the next 2 months after reporting of a glanders positive case. At the field level, a lack of enough manpower, personal protective equipment, sample collection materials, and sample transport mechanisms could be the reason behind the technical constraint (4). These constraints may be reduced by the distribution of responsibilities among multiple government organizations, sufficient financial support, a chip-based animal identification system, the provision of supporting staff in a glanders outbreak zone, an adequate supply of personal protective equipment and consumables for sample collection, and periodic revisions of compensation. Organization of workshops and meetings on the ethical and moral responsibilities of controlling outbreaks can also be helpful in increasing hierarchal harmony.

In our earlier study, we observed that only18% of in-contact humans from *B. mallei*-infected premises were surveyed for human glanders, and the number of human samples tested was lower in comparison to the prevalence of equine glanders in the country. In this study, 7.2% of the sampled participants were human health workers, and given that glanders is a highly contagious Zoonotic disease and a potent bio-warfare agent, intersectoral collaboration is a must in controlling this disease. Human glanders is perhaps under recognized and under reported and it needs more attention from health care workers. This data highlights the gap in the collaboration of veterinary and human health workers in tackling the *B. mallei* infection.

There are no vaccines or specific treatment regimen is available against glanders. However, vaccine experiments in non-human primates and laboratory animal models using glycol-conjugated or gold nanoparticle-based subunit vaccine showed partial protection against *B. mallei* infection (22, 23). Successful eradication and control of glanders can only be achieved by combining highly sensitive and specific testing methods with effective culling strategies. Close cooperation between animal husbandry authorities and equine keepers, as well as the strict compliance to biosafety approaches in animal holdings are essential (24). Most of the participants stressed that timely diagnosis, proper disposal of glanders infected animals and sterilization of in-contact premises are most important in controlling the *B. mallei* infection.

## 5 Conclusion

This study reveals that veterinarians need to be educated on governmental policies and guidelines on equine glanders with regular training and awareness programs. Sensitization of equine keepers regarding the early signs of glanders and prevention of diseases needs to be conducted on a regular basis. For this purpose, distribution of Information Education and Communication (IEC) print materials (leaflets, pamphlets and posters) on glanders in regional languages and social media can be used. One of the limitations of the present study was uneven distribution of respondents from glanders affected states. Therefore, a greater number of veterinarians from each state of the country may be included in future study. This may help in better implementation of glanders surveillance and control activities. Inter-sectoral coordination needs to be intensified to investigate human glanders.

## Data availability statement

The raw data supporting the conclusions of this article will be made available by the authors, without undue reservation.

## Ethics statement

Ethical review and approval was not required for the study on human participants in accordance with the local legislation and institutional requirements. Written informed consent from the participants OR participants’ legal guardian/next of kin was not required to participate in this study in accordance with the national legislation and the institutional requirements.

## Author contributions

AR: Data curation, Formal analysis, Writing – original draft. AP: Formal analysis, Writing – original draft. SK: Methodology, Writing – review & editing. BT: Funding acquisition, Investigation, Resources, Writing – review & editing. HT: Conceptualization, Methodology, Writing – review & editing. HS: Conceptualization, Formal analysis, Project administration, Writing – review & editing.

## Footnotes

^1^ https://pashudhanharyana.gov.in/sites/default/files/documents/Animal_health/NAP_Glanders_June_2019.pdf
