# Supplementary material for: Knowledge, awareness and perception about equine glanders among veterinarians and medical professionals in India
Source: Front Vet Sci. 2024 Mar 14;11:1334485. doi: 10.3389/fvets.2024.1334485 (PMC10976519; doi:10.3389/fvets.2024.1334485)
Supplement: Supplementary file 1 [file Data_Sheet_1.doc]

ICAR- National Research Center on Equines, Sirsa Road Hisar-125 001 (Haryana)

**KAP survey among the veterinarians regarding Glanders**

**Tick most suitable option(s). More than one option may also be chosen if it deems fit.**

| Name: ______________________ Highest Qualification & Discipline: ____________________ | | |
| --- | --- | --- |
| Present Post: ____________ Length of Service: ______________ Place of Posting: _________ | | |
|  | **Section 2 Knowledge test** |  |
|  | Glanders is a highly communicable disease of horses, mules and donkeys. | Yes/ No |
|  | It is a notifiable disease of equines caused by _________________________ (write agent name) | |
|  | Horses tend to be chronically affected, whereas donkeys and mules develop acute form. | Yes/ No |
|  | Clinical signs of Glanders include serial development of ulcerating nodules that are most found in the upper respiratory tract, lungs, and skin. | Yes/ No |
|  | Glanders is a zoonotic disease and can be used as potential biological weapon | Yes/ No |
|  | Among human, the disease is usually acquired through direct skin or mucous membrane contact with infected animal’s secretion/excretion/tissues. | Yes/ No |
|  | Is there any vaccine available for prevention and control of Glanders in India or elsewhere? | Yes/ No |
|  | What is the Act currently in vogue in India for control of infectious disease like glanders? (write the name) ______________________________________________________________________ | |
|  | Have you come across any case of glanders in your state or any part of India? | Yes/ No |
|  | Where is the National Referral Laboratory for glanders in India? _________________________ | |
|  | Will you suggest organization of equine fair in the event of glanders outbreak? | Yes/ No |

**Section – 1 Profile characteristics**

| **1. The major forms of Glanders are:**  i) Nasal  ii) Pulmonary  iii) Cutaneous  iv) All of the above | **2. Glanders may be diagnosed based on:**  i) Clinical signs  ii) ELISA  iii) Complement fixation test  iv) All of the above |
| --- | --- |
| **3.The major sources of transmission of Glanders are**  i) Ingestion of food or water contaminated with nasal discharges of carrier animals  ii) Ingestion of meat from affected horses  iii) Insect bites  iv) Inadequate nutrition and unhygienic conditions | **4. The major risk factors of spread of Glanders are:**  i) Sharing of feed and water troughs  ii) Lack of awareness and knowledge of owners about symptoms of disease  iii) Improper disposal of glanders affected carcass  iv) All of the above |
| **5. OIE recommended diagnostic test of glanders for international trade of equines in:**  i) Isolation and identification of the causative agent  ii) Cell- mediated immunity tests  iii) Complement fixation test  iv) ELISA | **6. Glanders can be prevented and controlled by:**  i) Early detection and elimination of affected animals  ii) Complete quarantine of positive animals before elimination  iii) Surveillance of in contact animal  iv) All of the above |
| **7. General biosafety precaution to be followed during sample collection from a suspected case of glanders includes wearing**  i) Gloves & face mask  ii) Disposable apron  iii) Goggles  iv) Boots/shoe cover  v) All of the above | **8. What is the present control policy of Glanders in India**  i) Quarantine and treatment of infected animals  ii) Treatment of positive animals and vaccination of in-contact animals  iii) Elimination of positive animals and surveillance of in-contact animals  iv) None of the above |
| **9. Following disinfectants may be used for decontamination of infected premises**  i) Sodium hypochlorite (500 ppm),  ii) Glutaraldehyde (2%)  iii) Benzalkonium chloride (2000ppm)  iv) All of the above | **10. On the zoonotic point of view sample should be collected from susceptible in-contact humans including**  i) Equine handlers  ii) Vet Officers  iii) Vet Assistant  iv) Farriers  v) All of the above |
| **Section – 3 Awareness test** | |
| **11. What would be your line of action if you come across any case of Glanders**:  i) Inform the concerned authorities  ii) Collect samples and sent to lab for confirmation  iii) Quarantined the animal till lab confirmation  iv) Elimination of the animal if confirmed report received | **12. What samples would you like to collect for diagnosis of glanders?**  i) Blood samples  ii) Nasal swab  iii) Nodule swab  iv) All of the above |
| **Section – 4 Perception test** | |
| **13. What factors do you perceive that Glanders has not been yet controlled in your state?**  i) It is not a priority disease in my state  ii) Lack of focus/ seriousness of field veterinarians for this disease due to fear of infection  iii) Owners hesitate to disclose the symptoms  iv) Lack of knowledge of field veterinarians regarding policy | **14. What type of constraints you generally face in reporting of Glanders and sending the samples to central laboratories**  i) Administrative  ii) Technical  iii) Financial  iv) Noncooperation and unwillingness by the superiors  v) None of the above  vi) All of the above |

**15. What specific suggestions and constraints (maximum 3) would you like to make about role of veterinarians in controlling Glanders?**
